# Optical properties, spectral, and lifetime measurements of central nervous system tumors in humans

**DOI:** 10.1038/s41598-017-14381-1

**Published:** 2017-10-25

**Authors:** F. Poulon, H. Mehidine, M. Juchaux, P. Varlet, B. Devaux, J. Pallud, D. Abi Haidar

**Affiliations:** 1 IMNC Laboratory UMR 8165-CNRS/IN2P3, Paris-Saclay University, Orsay, 91405 France; 2 Neuropathology Department, Sainte-Anne Hospital, Paris, 75014 France; 3 IMA BRAIN, INSERMU894, Centre de Psychiatrie et de Neurosciences, Paris, France; 4 Neurosurgery Department, Sainte-Anne Hospital, 75014 Paris, France; 5 Paris Descates University, Paris, France; 6 Université Paris Diderot, Sorbonne Paris Cité, F-75013 Paris, France

## Abstract

A key challenge of central nervous system tumor surgery is to discriminate between brain regions infiltrated by tumor cells and surrounding healthy tissue. Although monitoring of autofluorescence could potentially be an efficient way to provide reliable information for these regions, we found little information on this subject, and thus we conducted studies of brain tissue optical properties. This particular study focuses on the different optical quantitative responses of human central nervous system tumors and their corresponding controls. Measurements were performed on different fixed human tumoral and healthy brain samples. Four groups of central nervous system tumors (glioblastoma, diffuse glioma, meningioma and metastasis) were discriminated from healthy brain and meninx control tissues. A threshold value was found for the scattering and absorption coefficient between tumoral and healthy groups. Emission Spectra of healthy tissue had a significant higher intensity than tumoral groups. The redox and optical index ratio were thenn calculated and these also showed significant discrimination. Two fluorescent molecules, NADH and porphyrins, showed distinct lifetim values among the different groups of samples. This study defines several optical indexes that can act as combinated indicators to discriminate healthy from tumoral tissues.

## Introduction

The success of oncological surgery, the most widely used curative treatment for solid tumor whatever its histopathological type, is based on the accurate identification of the tumor’s boundaries in order to achieve a complete tumor resection. For tumors of the central nervous system, the goals of oncological surgery are identical but their realization is made more difficult by their infiltrating character, especially the diffuse gliomas, within a highly eloquent organ. The main challenge of any neurosurgical oncological intervention is to define the limits of the resection while optimizing the onco-functional balance^1^. Resection is based on the limits of tumor infiltration, which should be removed, and on the identification of eloquent brain areas, which should be respected. The actual identification of the tumor infiltration at the cellular scale is not possible intraoperatively and requires the development of an efficient and reliable intraoperative imaging tool, based on an imaging database of the main tumors of the central nervous system (diffuse gliomas, metastases, meningiomas, and healthy tissues).

The development of an intraoperative probe/device should be accompagnied by the construction of a large database of endogenous fluorescence response of tumor tissues. This intraoperative tool, in association with knowledge on collected optical response, will lead to an “optical biopsy” giving a real time result, and providing additional relevant morphological and physiological information during surgery, which may guide the surgical resection.

The optical properties of biological tissues have a major importance in several medical applications for diagnosis and therapy^2^. Knowing the optical properties of brain tissues results in quantitative information^3^, which allows the optimization of imaging techniques and the possibility of modeling the light path, the fluorescence distribution, the penetration depth and the possible interaction between fluorophores in the tissues. These parameters are related to the density and the distribution of sizes of the ultrastructure of a tissue, thus allowing the characterization of the tissues, particularly the differentiation of tumoral tissues from healthy ones–in other words, detection of the evolution of a pathology. Current knowledge of optical properties of healthy and tumoral brain tissues is insufficient and does not exist for different tumoral varieties^4–6^. There is not only a lack of data in the visible spectral range, especially for 405 nm excitation wavelength, which is optimal to excite different endogenous fluorophores such as nicotinamide adenine dinucleotide (NADH), flavins (FAD), lipopigments and porphyrins, but also a lack of results on human brain tissues^2,3^. In contrast there exists a large literature on rats, mice, pigs and kidney tissue^7,8^.

This lacuna in the literature, as well as surgeon’s needs, motivates our exploratory study on the optical coefficients, absorption, scattering and anisotropy. These optical parameters are required not only to correctly understand the signal from endogeneous fluorescence of tissue, but to have real time quantitative data about the nature of the tissue. The most suitable and growing technique is to track spectral response. A few research teams have already started to work on spectral emission of healthy or tumoral brain tissue^9^, but as of now none have really tackled a large number of types of brain tumors.

Our previous study on the grade of malignancy of meningiomas in adults^10^ gave us interesting results through the use of spectral response to dicriminate among tissues. Here, the same technique will be applied to a larger cohort and will also be used to track metabolic changes. Indeed, we can find in the literature that some groups have started to calculate the ratios of the contribution of molecules in the emission spectra to track the metabolic reaction^11–13^. In addition, another quantitative response can now be measured due to progress in nonlinear optics and access to femtosecond pulsed laser, and that is fluorescence lifetime^14,15^. This measurement is influenced not by the concentration of fluorescent molecules, but by the conformational or environmental changes surrounding the fluorescent molecules.

A study of the optical properties, the spectral response, and lifetime of the endogeneous fluorescence on brain tissue, will allow an understanding of the evolution of the hyperstructure, the metabolic process and environment transitionning from a healthy region to a tumoral region. In this paper, we studied a wide cohort of brain tissues, including four tumor types and a control group (epileptic cortex). To span the wider range of tumor types, we chose primary (glioblastoma, meningioma and diffuse glioma) and secondary (metastasis) tumors of the central nervous system. Several optical properties were looked at, using integrating-sphere techniques and employing an inverse Monte Carlo technique. The absorption, scattering and anisotropy coefficients were measured using 405 and 430 nm excitation wavelengths. Moreover, spectral and lifetime fluorescence measurements were acquired on these tissues using 405 nm and 375 nm excitation wavelengths. The 405 nm excitation wavelength was used to excite and efficiently collect the fluorescence signal of five endogenous fluorophores: NADH, FAD, lipopigments, porphyrins and chlorins. The 375 nm excitation wavelength was used to excite efficiently the NADH and the FAD. The 405 and 430 nm excitation wavelengths were used in the integrating sphere technique to study the effect of the wavelength on the optical coefficients. Through the analysis of all these quantitative data, we were able to define several discriminatory indicators between control and tumor samples and also we were able to discriminate several tumor types and grades of malignancy.

## Results

**Figure 1:**
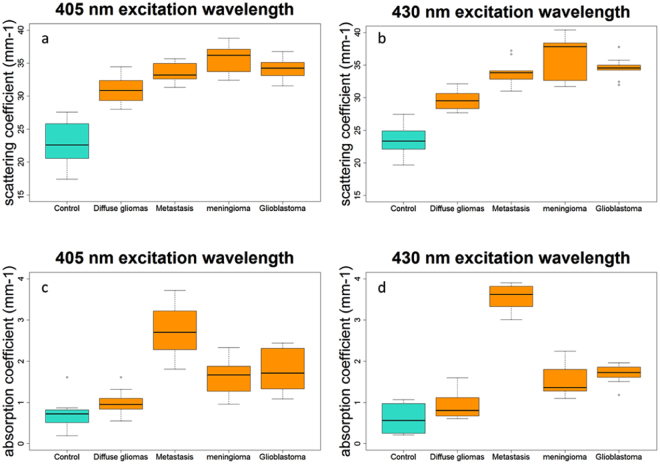
Distribution of scattering coefficient values for tumoral and healthy control tissues excited with 405 nm (**a**) and 430 nm (**b**), and of absorption coefficient using 405 nm (**c**) and 430 nm (**d**) excitation wavelengths.

Figure 1 summarizes the scattering (µ_s_) and absorption (µ_a_) coefficient for different types of brain tissues at two different excitation wavelengths.

Figure 1a,b show the scattering coefficient results for healthy (control) and tumoral (diffuse gliomas, metastasis, meningioma and glioblastoma tissues). The tumoral tissues present a significantly (p < 0.001) higher scaterring coefficient Fig. 1c,d represent the absorption coefficient of tumoral and control tissues. Metastasis, meningioma and glioblastoma are significantly (p < 0.001) different from control tissues. Diffuse gliomas other than glioblastoma have an absorption coefficient not significatly different to those of control tissues (p = 0.09).

**Figure 2:**
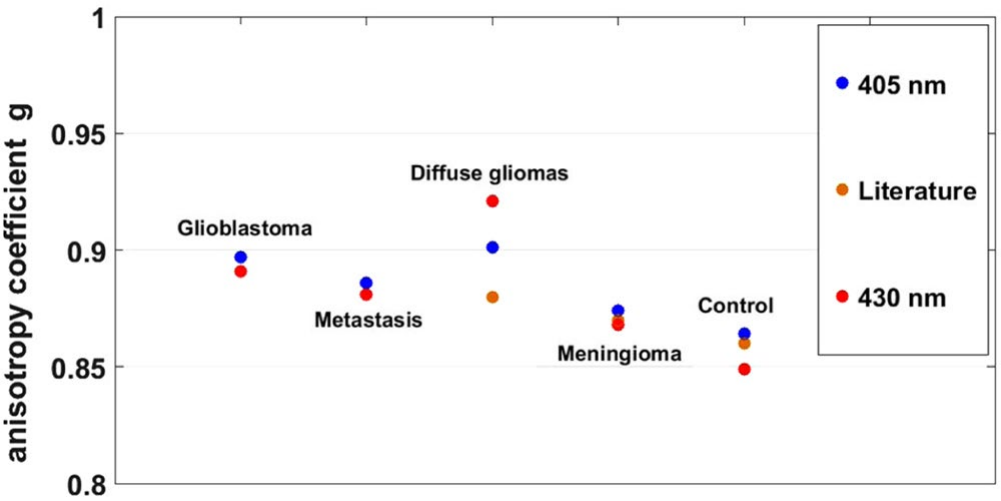
Average of anisotropy coefficient g, under 405 and 430 nm wavelength excitation and compared to the g values found in literature.

The anisotropy coefficient could also be calculated for 405 and 430 nm excitation wavelength. The results for the different groups are plotted in Fig. 2. Three groups of tissues had already been examined in the literature and allowed us to compare our values, the meningioma with a g = 0.87^3^ where we found 0.87 and 0.86, the control tissue with a g = 0.86^3^ where we found 0.85 and 0.865 and the diffuse gliomas with a g = 0.88^3^ where we found 0.90 and 0.20. Our values were in line with the previous literature which validates our set-up and protocols. We also measured two other types of tumor that were not tackled before, adding information to the existing literature. A trend can be extracted from the value, ie, that the tumoral tissues tend to have higher g value than the control tissues. However, this is not statistitically significant.

Having seen these differences in the fundamental optical properties of the tissue, we needed to look at other measurements of the endogeneous fluoresence, that could be implemented more easily *in-vivo* and could also give an insight on the metabolic state of the tissue. Through a first bi-modal device developped in our group^16^, we measured consecutively the spectral and fluorescence lifetime response from tissues.

**Figure 3:**
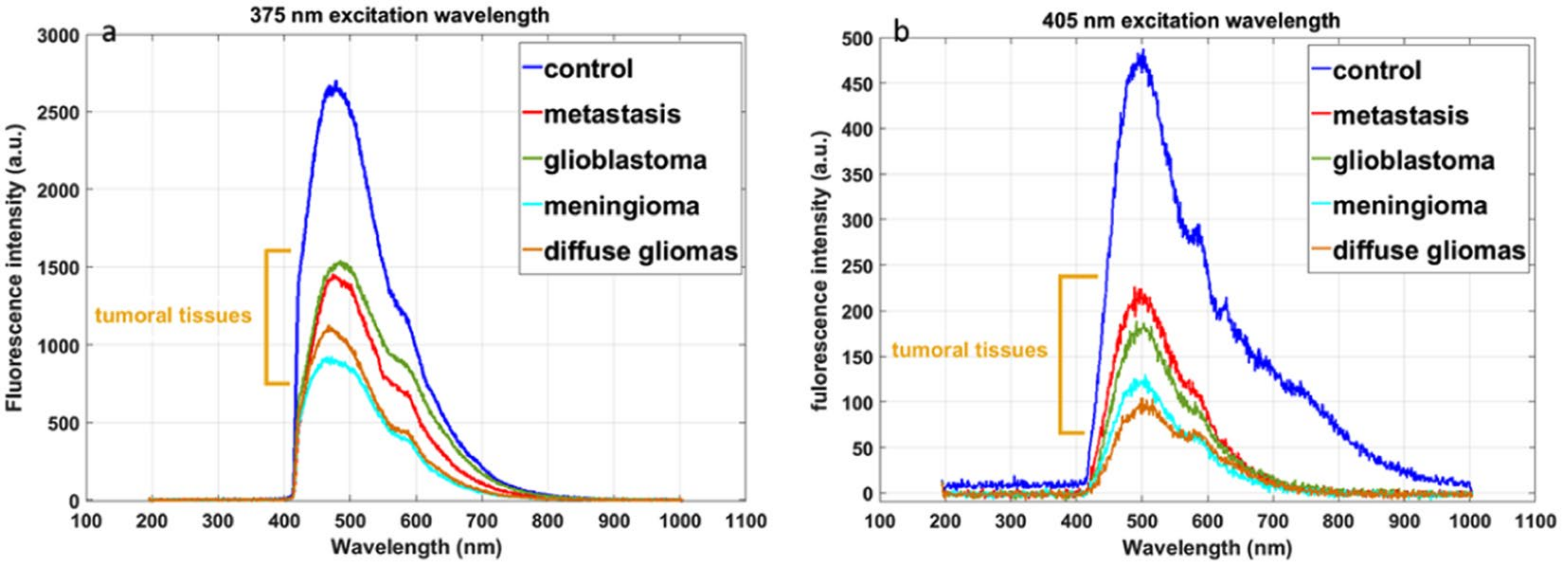
Fluorescence spectra of all tissues excited with 405 nm wavelength (**a**) and 375 nm (**b**).

Figure 3 presents the emitted fluorescence of each type of tissue at the 375 and 405 nm excitation wavelengths. The shapes of the spectra change with 405 nm excitation wavelength. At this wavelength, we were able to excite five endogenous molecules. With 375 nm, we efficiently excite NADH and FAD only, thanks to their higher absorption cross section at this excitation wavelength. We can underline a difference in the fluorescence intensity between each type of tissue. The gap between control and tumoral tissues was significant and encouraging. At both wavelengths, the healthy tissue has significantly higher intensity than tumoral tissue. This is explained by the lower absorption and scattering coefficient in healthy tissue that results in more fluorescence emitted and collected by our set-up.

Every spectra is the sum of emission spectrum of five fluorphores: NADH, FAD, lipopigments, porphyrins and chlorins.

In addition, by calculating the integral under the curve of the emission spectra of NADH, FAD and the porphyrins, using a Matlab program developed by our team^17^, we obtained two different ratios under 405 nm and 375 nm excitation wavelengths^11,18^.

$${\rm{Under}}\,{\rm{375}}\,{\rm{nm}}\,{\rm{excitation}}\,\mathrm{wavelength},\,{\rm{we}}\,{\rm{calculated}}\,{\rm{the}}\,{\rm{redox}}\,{\rm{ratio}}\,(\mathrm{ROx})=\frac{{\rm{FAD}}-{\rm{NADH}}}{{\rm{FAD}}+{\rm{NADH}}}$$

$${\rm{Under}}\,{\rm{405}}\,{\rm{nm}}\,{\rm{excitation}}\,\mathrm{wavelength},\,{\rm{we}}\,{\rm{calculated}}\,{\rm{the}}\,{\rm{optical}}\,{\rm{index}}\,{\rm{ratio}}\,(OIR)=\frac{{\rm{NADH}}}{{\rm{Porphyrins}}}$$

**Figure 4:**
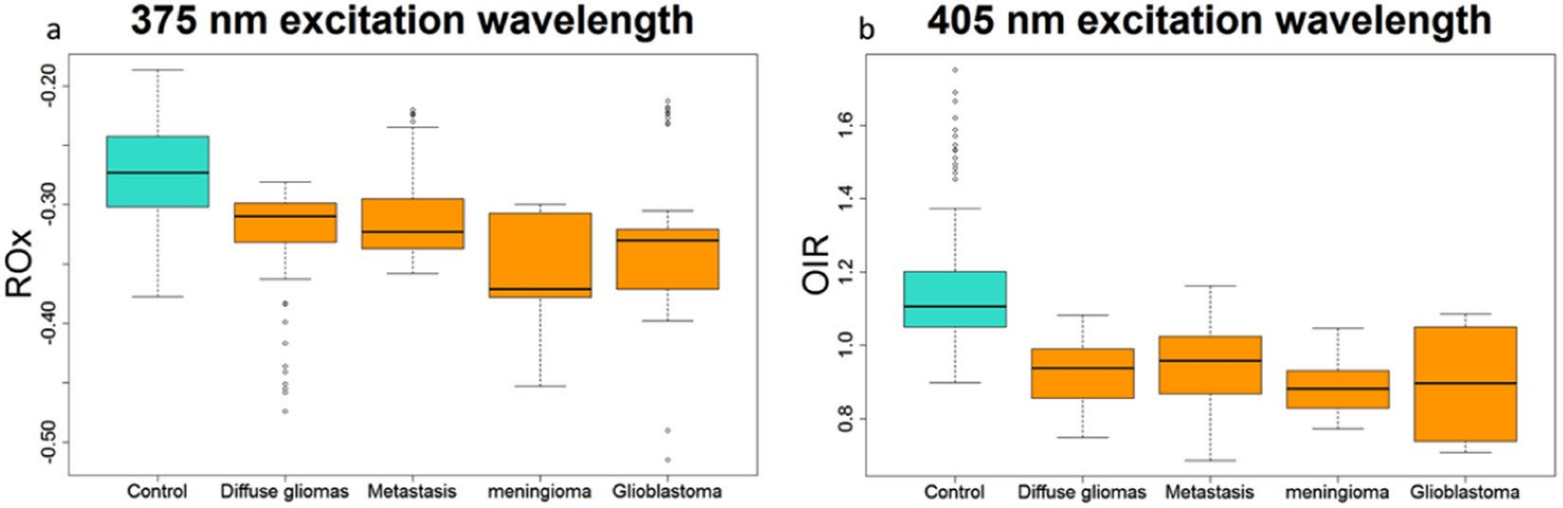
(**a**) Variation of the ROx report of all tissues excited with 375 nm and (**b**) Variation of the NADH/ Porphyrins of all tissues excited with 405 nm.

At 375 nm, the calculated ratio has negative values because NADH is more present in brain tissues than FAD at the excitation wavelength^17,19^. As shown in Fig. 4a, the healthy tissues have a higher ROx than tumor tissues (p < 0.001), which is inline with the literature^11^.

At 405 nm, the Fig. 4b also shows a significative difference (p < 0.001) tumoral and healthy tissues. Similar results were obtained on bladder tumor tissues^19^.

To complete our study, we also collected data from a promising quantitative technique, the fluorescence lifetime. As previously done, we used two excitation wavelengths, 375 nm and 405 nm. We had a closer look at two molecules emitting endogeneous fluorescence. This choice was motivated by our previous study on a cohort of fresh samples that highlights the changes in NADH and our study on brain rats that shows a variation in porphyrins^17,19^.

**Figure 5:**
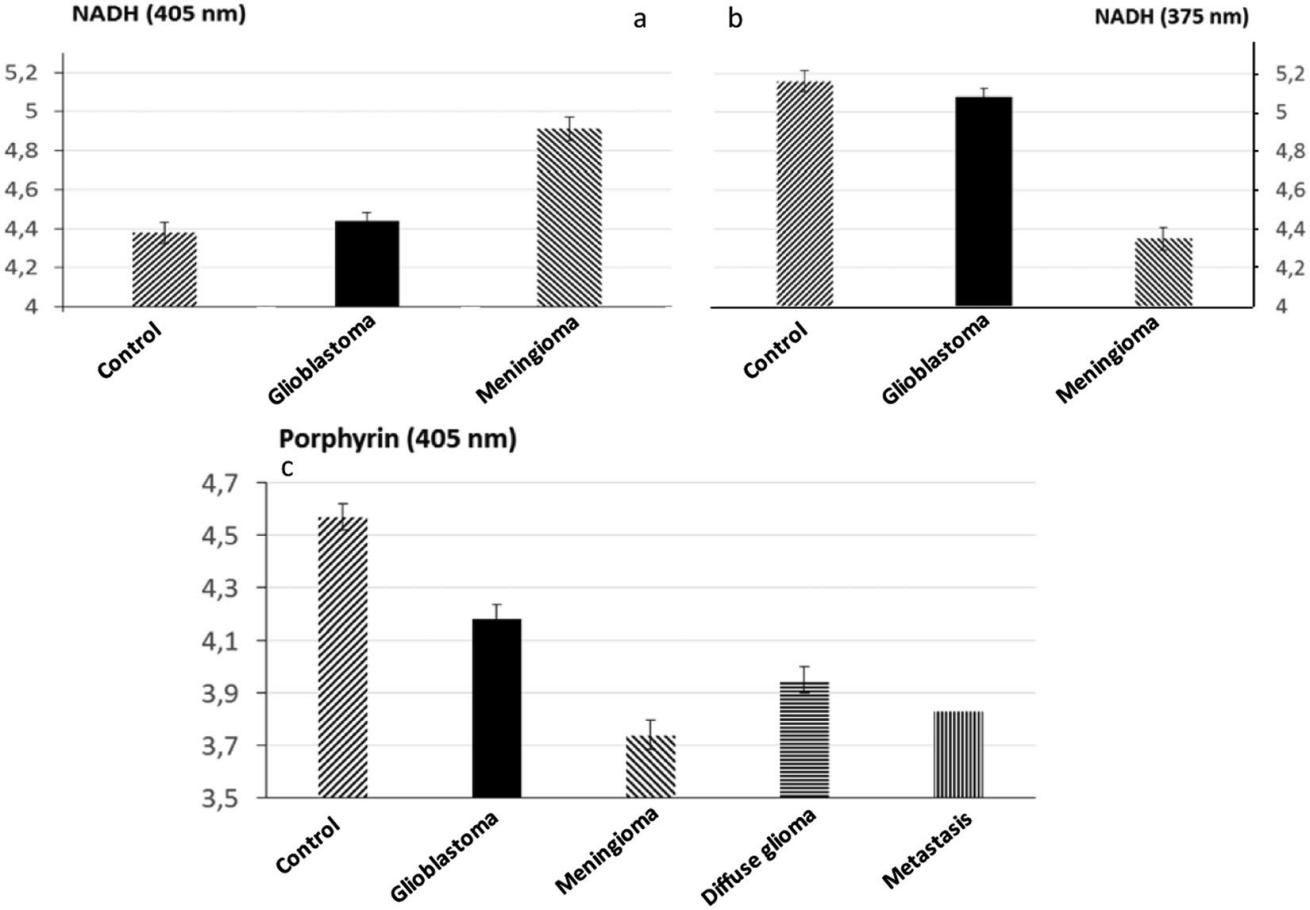
Comparaison of mean fluorescence lifetime in different types of tissues. Evolution of NADH lifetime at 375 nm (**a**) and 405 nm(**b**) in three types of tissues (control, glioblastoma, Meingioma). Evolution of porphyrins lifetime at 405 nm between control, glioblastoma and meningioma (**c**), between diffuse gliomas and control (**d**) and between metastasis and control (**c**).

In Fig. 5a,b we see that at both excitation wavelengths, the NADH presents a difference in lifetime between glioblastoma, meningioma, and control samples. Nevertheless, at 405 nm excitation wavelength, the control has a shorter lifetime than healthy tissue, where at 375 nm the control has a longer lifetime. The meningioma is very well distinguished from the control tissue (p = 0.002 at 405 nm; p = 0.004 at 375 nm).

**Figure 6:**
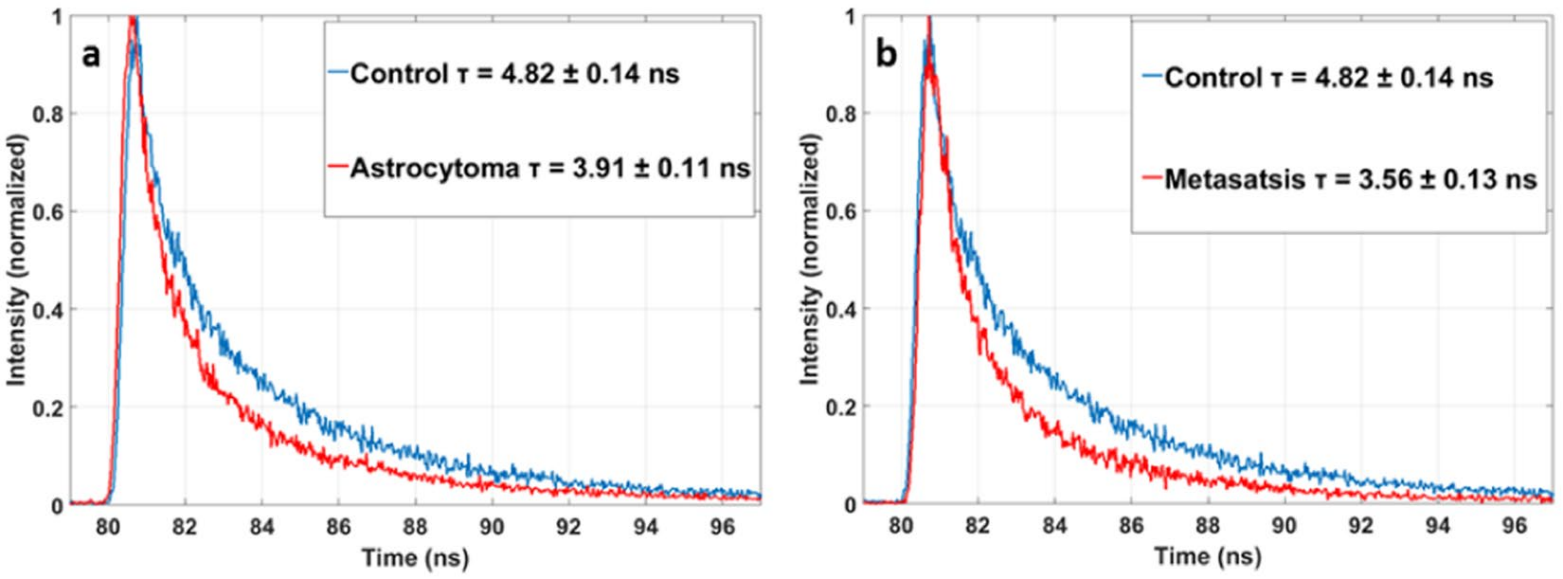
Histogram of lifetime fluorescence decay of the porphyrin component with a 405 nm excitation between astrocytoma and control (**a**) and between metastasis and control (**b**).

In Fig. 5c the results for the porphyrin, well excited at 405 nm are presented in all the tumoral tissues (Glioblastoma, Meningioma, Diffuse glioma and Metastasis) a significantly shorter lifetime than the control tissue, respectively with a p-value of p = 0.05, p = 0.001, p = 0.02 and p < 0.001. The Fig. 6 illustrates this difference between a tumoral tissue and a control looking at the histogram of fluorescence lifetime decay.

## Discussion

In this study, an integreting sphere was used to measure the optical parameters of sample from healthy brain tissues and from tumors of the central nervous system of human patients. Different tumor tissues were selected, from primary or secondary tumors and with different grades of malignancy.

We measured the optical properties with different excitation wavelengths. The results improved our understanding of the evolution of the absorption coefficient and scattering coefficient on different types of tissues. We could observe a trend from these results, the scattering coefficient of healthy tissues is lower than those from the different tumor tissues. This difference can be explained by the fact that tumor tissues have more collagen fibers and a stronger vascularisation than healthy ones, thus the source of stronger scattering. A threshold can be determined to discriminate between healthy and tumorous. Regarding this coefficient a tissue with µ_s_ < 26 is healthy and with a µ_s_ > 29 is tumoral. In the interval where µ_s_ ∈ [26, 29] no conclusion can be drawn on the tissue nature. The absorption coefficent also discriminates meningioma, glioblastoma and metastasis from healthy tissue. It can be explained by the stronger vascularization in tumoral tissue. In addition, the higher the grade of malignancy, the more the chromatin is condensed, so the absorption is higher (seen in the evolution µ_a_ of from healthy, diffuse glioma to glioblastoma). The obtained values for the different coefficient were closed to the literature for the tissues studied in similar conditions or when applied on animals^3^.

The spectral response confirmed the conclusions made on the optical properties. The evolution of spectra from one tissue to another one is in line with the observed change in absorption and scattering coefficient. These spectral measurements have been established in a “fibered” configuration to be as close as possible to clinical intraoperative configuration. These spectral results gave us another thechnique with which to look at the discriminating indicator found with optical coefficients.

We further exploited the spectral response by looking at the metabolic ratio in order to get a more robust indicator of tissue nature. In our previous work^17^, we had noticed that in rat brain tissues the porphyrin showed different responses in terms of fluorescence lifetime depending on tissue nature. Observing the important presence of porphyrins in the human brain tissue spectral response, we wanted to exploit our knowledge of rats and look at the ratio between NADH and porphyrins to obtain more discriminatory information. This ratio will link the metabolic and vascular aspect of tissues and consequently provide decisive information.

To finalize the sudy and obtain more quantitative data, the fluorescence lifetime has also been measured with the intraoperative device configuration using 375 nm and 405 nm as excitation wavelengths. Results are interesting and showed the sensitivity “of the measure to the nature and state of conservation of tissues”. If we compare these results on fixed tissues to the ones previously obtained on fresh tissues with same the same set-up^19^, we observe a variability in results. For example for the NADH in cortex-control fixed tissue the mean lifetime is 4.44 ns, whereas for the fresh tissue in our previous article the mean lifetime is 3.38 ns. We can also conclude, as in the rat studies, that porphyrin is a strong indicator of tissue nature, giving us a discriminatory response between tumoral and the healthy tissues and among the tumors (glioblastoma, diffuse glioma, metastasis and meningioma), we also went further in this study showing that NADH had also a fluorescence lifetime different from one tissue to another, significativly discriminating meningioma from healthy tissue.

We have aimed to present an original study, focused on the autofluorescencte response of healthy and tumoral brain tissues observed with different modalities, either in a fiber configuration or in a integrating sphere set-up. All these techniques allowed to establish a group of discriminating indicators between healthy and tumoral. To our knowledge, such a study on a substantial cohort of human samples has never been performed. As a next step, we wish to expand our cohort to tumor margins, samples where the concentration in tumor cells is very low, in order to test the reliability of the indicators we demonstrated here.

## Materials and Methods

### Samples

An approval of the Sainte-Anne Hospital – University Paris Descartes Review Board (CPP Ile de France 3) was obtained for this study in collaboration with the Neurosurgery and the Neuropathology Departments of the Sainte-Anne Hospital (S.C.3227). All the following methods were performed in accordance with the relevant guidelines and regulations from this protocol and a informed consent was obtained from all participants and/or their legal guardians.

A cohort of twenty height samples was used, the repartition is resumed in the Table 1. A group of ten healthy cortex samples was obtained from epileptic surgery and compared to four different groups of central nervous sytem tumors. The tumoral cohort contains four Isocitrate Deshydrogenase (IDH) wildtype glioblastomas, five IDH-mutated diffuse gliomas, five meningiomas and five carcinoma metastases originating from different part of the organism.

**Table 1:** Summary of the cohort used in the paper.

**Healthy**		**10**
**Metastasis**		**5**
	*Thyroid carcinoma*	*1*
	*Colloid adenocarcinoma*	*2*
	*Primary renal carcinoma*	*1*
	*Cell lung carcinoma*	*1*
**Diffuse Glioma**		**5**
**Glioblastoma**		**4**
**Meningioma**		**4**

Once received from the hospital, the samples were stored at −80 °C. Few hours before cutting they were put at −20 °C. Then, the tissues were cut at −18 °C into 200 µm and 600 µm slices using a cryostat (CM 1950, Leica Microsystems). After, tissues were fixed with ethanol at 100° and stored at 4 °C until the experiment. The slices of 200 µm were used in the integrating sphere set-up to measure the optical coefficients and the slice of 600 µm were use in the endoscopic set-up for spectral and lifetime measurements.

### Integrating sphere set-up

A standard set-up was used for the measurements of transmittance T(λ) and reflectance R(λ). It consists of an integrating sphere (model: IS200-4 thorlabs) including four identical ports, each port has a 12.7 mm diameter, and a fifth port with a 3 mm diameter used to collect light from the sphere to a spectrometer (HR2000-Ocean optics) using an optical fiber (QP60-600 µm diameter Ocean optics). The excitation was achieved with two laser diodes, emitting at 430 nm (LDH-P-C-430B, Picoquant Germany) and 405 nm (LDH-P-C-405B, Picoquant Germany) respectively with a maximal power of 5.1 mW and 1 mW respectively. To have a 1 mm laser beam diameter, a diaphragm was placed just after the laser diodes.

For collimated transmittance measurements, the integrating sphere is not used, three diaphragms were aligned in front of the laser, and an absorbant filter (OD = 2.3) is placed to reduce the laser intensity.

In each sample, five regions of interest (ROI) were selected in areas of similar visual apparence, and the transmittance, the reflectance and the collimated transmittance were measured. The average of these five measurements was used to determine the optical coefficients of the sample.

### Spectral and lifetime measurements

Details of this setup have been published elsewhere^16,19^. The exciation is performed with two pulsed diode lasers from Picoquant, emiting at 405 nm (LDH-P-C-405B, FWHM 60 ps, Picoquant-Germany) and 375 nm (LDH-P-C-375B, FWHM 45 ps, Picoquant-Germany). The diodes are controlled by a driver (PDL-808 “Sepia”, PicoQuant GmbH, Berlin, Germany). The repetition frequency used for this study is 40 MHz.

A single-core fiber with a 200 μm diameter, is used to bring the excitation to the sample. The signal is then collected through a single-core optical fiber with a 365 μm diameter. Then the collected signal goes through a long pass filter (SR420, Semrock) to cut the signal from laser reflection. For spectral measurement, a spectrometer (QEPro 6500, Ocean Optics, 1.5 nm spectral resolution over a 365-950 nm spectral range) was used. For lifetime measurements a filter wheel (FW102C, Thorlabs, Newton, USA) with five filters (Semrock, New York, USA, 450 ± 10 nm, 520 ± 10 nm, 550 ± 30 nm, 620 ± 10 nm and 680 ± 10 nm) in front a photomultiplier link to a TCSPC acquisition card (PMA 182 and Time Harp 200, Picoquant, Germany) was used.

### Data analysis

#### Optical properties

Reduced scattering (μ_s_’), absorption (µ_s_) and anisotropy coefficients (g) of the samples were obtained from measured values of the reflectance, transmittance and collimated transmittance using the Inverse Adding Doubling (IAD) algorithm developed by scott Prahl (http://omlc.ogi.edu/software/iad/)^20^. This algorithm solves iteratively the radiative transport equation until the numerical adjustment and the experimental values of reflectance, transmittance and collimated transmittance matches, it also takes the parameters of the sample, the laser beam and the integrating sphere used into account to find the optical coefficients desired.

Noting that ${{\rm{\mu }}}_{{\rm{s}}}^{^{\prime} }$ is the reduced scattering coefficient, we will deduce the scattering coefficient µ_s_ using the equation below

$${{\rm{\mu }}}_{{\rm{s}}}={{\rm{\mu }}}_{{\rm{s}}}^{^{\prime} }/(1-{\rm{g}})$$

According to the literature^2,6^, the refractive index of human brain tissues is between 1.33 and 1.53. We have considered that the refractive index is n = 1.4 for all samples^2^.

#### Spectral analysis

At the excitation of 375 and 405 nm five fluorophores are excited: NADH, FAD, lipopigments, porphyrins and chlorines. The measured spectra present the sum of the emission spectrum of these endogenous fluorophores.

Spectral data are processed from a Matlab program developed previously by our team and already used in a previous publication [1]. Using this program we can determine the contribution of each molecule by adjusting the measured data to the equation below

$${{\rm{S}}}_{{\rm{total}}}(\lambda )={\sum }_{{\rm{i}}=1}{({\rm{f}}}_{{\rm{i}}}.{{\rm{S}}}_{{\rm{i}}}({\rm{\lambda }}))$$

where: S_total_ (λ): measured spectra,

i: the fluorophore,

S_i_ (λ): emission spectra of the fluorophore I,

f_i_: multiplying coefficient.

NADH spectra is adjusted by a spectra obtained from a previous experiment done by our team on rats at 375 nm^17^. FAD spectra is adjusted by a spectra taken from the literature^21^. The other spectras of Lipopigments, Porphyrins and Chlorins are given by Gaussian adjustment.

#### Lifetime analysis

The measured fluorescence decay curves obtained are adjusted by a mono-exponential fit using FluoFit software (Picoquant,Germany), which allows us to extract the fluorescence lifetime of the curve. Two criteria are taken into account to validate the fit: χ2 must be −1.2 < χ^2^ < 1.2, and the residuals must have a distribution around 0 in an interval of [−4, 4].

#### Staistical analysis

Optical coefficients(scattering and absorption) and fluorescence lifetime results were evaluated using a one-way analysis of variances (ANOVA). If the ANOVA was statistically significant, a post-hoc t-test was performed. A probability value (p) < 0.05 was considered statistically significant.

## References

[1] Pallud J, Dezamis E (2017). Functional and oncological outcomes following awake surgical resection using intraoperative cortico-subcortical functional mapping for supratentorial gliomas located in eloquent areas. Neurochirurgie..

[2] Bevilacqua F (1999). *In vivo* local determination of tissue optical properties: applications to human brain. Appl. Opt..

[3] Yaroslavsky AN (2002). Optical properties of selected native and coagulated human brain tissues *in vitro* in the visible and near infrared spectral range. Phys. Med. Biol..

[4] Cheong WF, Prahl SA, Welch AJ (1990). A review of the optical properties of biological tissues. IEEE J. Quantum Electron..

[5] Bashkatov AN, Genina EA, Tuchin VV (2011). Optical Properties Of Skin, Subcutaneous, And Muscle Tissues: A Review. J. Innov. Opt. Health Sci..

[6] Jacques SL (2013). Optical properties of biological tissues: a review. Phys. Med. Biol..

[7] Swartling J, Pålsson S, Platonov P, Olsson SB, Andersson-Engels S (2003). Changes in tissue optical properties due to radio-frequency ablation of myocardium. Med. Biol. Eng. Comput..

[8] Solonenko M (2002). *In vivo* reflectance measurement of optical properties, blood oxygenation and motexafin lutetium uptake in canine large bowels, kidneys and prostates. Phys. Med. Biol..

[9] Croce, A. C. & Bottiroli, G. Autofluorescence spectroscopy and imaging: a tool for biomedical research and diagnosis. *Eur*. *J*. *Histochem*. **58**, (2014). 10.4081/ejh.2014.2461 PMC4289852 25578980

[10] Zanello, M. *et al*. Multimodal optical analysis of meningioma and comparison with histopathology. *J*. *Biophotonics* n/a-n/a doi:10.1002/jbio.201500251 (2016). 10.1002/jbio.201500251 26871683

[11] Cicchi R (2010). Time- and Spectral-resolved two-photon imaging of healthy bladder mucosa and carcinoma *in situ*. Opt. Express.

[12] Skala MC (2007). *In vivo* multiphoton microscopy of NADH and FAD redox states, fluorescence lifetimes, and cellular morphology in precancerous epithelia. Proc. Natl. Acad. Sci..

[13] Papayan G, Petrishchev N, Galagudza M (2014). Autofluorescence spectroscopy for NADH and flavoproteins redox state monitoring in the isolated rat heart subjected to ischemia-reperfusion. Photodiagnosis Photodyn. Ther..

[14] Butte PV (2011). Fluorescence lifetime spectroscopy for guided therapy of brain tumors. NeuroImage.

[15] Berezin MY, Achilefu S (2010). Fluorescence Lifetime Measurements and Biological Imaging. Chem. Rev..

[16] Ibrahim A (2016). Spectral and fluorescence lifetime endoscopic system using a double-clad photonic crystal fiber. Opt. Lett..

[17] Haidar DA, Leh B, Zanello M, Siebert R (2015). Spectral and lifetime domain measurements of rat brain tumors. Biomed. Opt. Express.

[18] Palmer, S. *et al*. Optical redox ratio and endogenous porphyrins in the detection of urinary bladder cancer: A patient biopsy analysis. *J*. *Biophotonics* doi:10.1002/jbio.201600162 (2016). 10.1002/jbio.201600162 27714989

[19] Zanello M (2017). Multimodal optical analysis discriminates freshly extracted human sample of gliomas, metastases and meningiomas from their appropriate controls. Sci. Rep..

[20] Taddeucci A (1996). Optical properties of brain tissue. J. Biomed. Opt..

[21] Barrio JR, Tolman GL, Leonard NJ, Spencer RD, Weber G (1973). Flavin 1, N 6 -ethenoadenine dinucleotide: dynamic and static quenching of fluorescence. Proc. Natl. Acad. Sci. USA.

